# Ascertaining the Inconsistency of AEC Students’ Perceptions and Behaviors Regarding Sustainability by Mixed Methods

**DOI:** 10.3390/ijerph182413274

**Published:** 2021-12-16

**Authors:** Yuanxin Zhang, Liujun Xu, Wei Wu, Zaijing Gong, Hashem Izadi Moud, Zhihua Luo

**Affiliations:** 1 School of Management, Guangzhou University, Guangzhou 510006, China; yuanxin@gzhu.edu.cn (Y.Z.); liujun@e.gzhu.edu.cn (L.X.); luozhihua@gzhu.edu.cn (Z.L.); 2 Department of Construction Management, California State University, Fresno, CA 93740, USA; weiwu@csufresno.edu; 3 Construction Management Department, U.A. Whitaker College of Engineering, Florida Gulf Coast University, Fort Myers, FL 33965, USA; hizadimoud@fgcu.edu

**Keywords:** sustainability, construal level theory, psychological distance theory, factorial experiment design, mixed methods

## Abstract

University students in architecture, engineering, and construction (AEC) are the main force and future leaders of the construction industry, and their values shape the model and direction of the industry’s future development. The construction industry is the largest contributor of waste and greenhouse gas emissions. However, there is an inconsistency between AEC university students’ perceptions and behaviors regarding sustainability, which has received little attention. This study attempts to shed light on the root causes of the inconsistency from the psychological perspective, incorporating construal level (CL) theory and psychological distance (PD) theory into situational settings of the experiment. We recruited 556 AEC students from 20 different universities to participate in data collection. Research findings revealed that PD has a significant influence on AEC students’ recycling behavior with variance in the effect of different dimensions, even though CL has no significant impact. Furthermore, findings show that spatial distance poses the greatest impact on AEC student recycling behavior, followed by information distance, temporal distance, experience distance, hypothetical distance, and social distance. This study contributes to the body of knowledge by introducing CL and PD into sustainability perception and behavior research in construction and has practical implications for universities with sustainability curricula in AEC.

## 1. Introduction

The annual output of municipal solid waste in China has exceeded 150 million tons and is continuously increasing at a rate of 8–10%, which makes China a major contributor of waste [1]. Although modern technology can reduce and treat harmful waste, with the continuous increase in waste production, disposal facilities are facing overloaded operations. As landfill capacity is limited and waste occupies a huge area, it generates conflicts between the number of humans and the amount of land in cities [2]. In addition, the high concentration of leachate infiltrating the soil, the emission of harmful gases into the air during incineration, and the odor that permeates during waste transportation and processing [2] have resulted in significant damage to the ecological environment, seriously affecting the lives of nearby residents [3]. Public distrust of waste treatment facilities has also made it difficult to select sites for disposal of urban domestic waste [4]. The conflicts between humans and accessible land, environmental issues, and other social conflicts are forcing humans to recycle, thereby reducing the amount of waste originally generated [5,6].

As a vital economic pillar, the construction industry is one of the main sources of waste [7], creating a considerable environmental impact [8]. Construction projects consume astronomical quantities of valuable natural resources [9,10] such as fresh water, wood, and minerals, and damage to vegetation has caused serious ecological imbalance and soil erosion [11]. Statistics from the Ministry of Housing and Urban-Rural Development of China show that, globally, more than 40% of energy consumption and 21% of greenhouse gas emissions originate from the construction industry. More than 50% of the raw material obtained in nature is used to construct various types of facilities and their ancillary apparatus—which consume about 50% of the global energy in processes such as construction and operation, air and light pollution, and electromagnetic pollution related to buildings—account for 34% of the world’s environmental pollution. Construction waste accounts for 40% of all waste generated by human activity [12]. Construction has been shown to be one of the important factors leading to an escalation of the environmental challenges facing the earth [13].

Although the World Commission on Environment and Development (WCED) has convened a large number of conferences related to sustainable development in order to solve problems in sustainable development, governments of various countries have also established special environmental protection departments, issued various policies, and made unremitting efforts in many aspects [14]. Yet studies have indicated that there are often significant differences in perceptions and behaviors associated with sustainable development [15,16,17,18,19]. Many people are highly aware of environmental protection subjectively, and objectively feel frequently threatened by environmental damage, but not all adjust their actions to address it. A recycling campaign has been in place in China’s big cities lately. Although only 0.9% of residents are not aware of it, the actual level of citizen involvement in recycling is low, especially in small cities [20]. Hence, although citizens’ awareness and concern regarding sustainable development continue to increase, the rate that environmental awareness is being converted into corresponding sustainable behaviors is still very low. The difference in people’s perceptions of sustainable development and behaviors associated with it suggests that recycling campaigns only raise awareness and do not affect behavior [21]. If things continue in this way, the marginal utility of advertising will decrease dramatically. Therefore, the difference in perception and behavior directly affects sustainable development and requires interference to change its course toward consistency between high perception and responsible behaviors.

Because the values of university students in architecture, engineering, and construction (AEC) can shape the mode and direction of industry development as well as affect the words and deeds of people around them in future, they are the backbone and future leaders of sustainable development. As university students are a highly educated group and are likely to be the elites of the society, they are most likely to take key leadership roles and can make lots of critical decisions in their job careers. Some of these decisions may be to determine how the construction industry evolves regarding sustainability and will therefore exert profound influence on the industry [22,23]. According to data from the China Statistical Yearbook in 2020, the number of students in colleges and universities nationwide was 32.85 million, and the number of graduates reached 7.97 million [24]. This indicates that, in the future, the main members of society will be replaced by university students. Furthermore, university students are in the critical period of establishing value systems and cultivating correct behavior habits. Their value and behavior habits are not only tied to their career development, they will also bring their own sustainable development behavior habits to all aspects of work and affect the sustainable development of all aspects of the construction industry. It is important to explore the critical influencing factors on their recycling behavior and make countermeasures to shape their value system. There is also a general agreement on the view that significant changes in individual behavior are required for society to move towards sustainability [25,26,27]. As a result, this study chooses university students as the subject to investigate what affects their perceptions and behaviors in sustainability from psychological perspective, because finding the critical influencing factors can offer a reference for the construction related programs of universities to adjust their curriculum and pedagogical practice so as to improve students’ sustainable perception and behavior, passing this into their future career. Subsequently, their improvement in sustainability can transform and upgrade the construction industry as a whole.

Construal level (CL) theory as a cognitive social psychology theory conceptualizes individuals’ psychological representations of cognitive objects or events into different levels of interpretation, showing the underlying logic individuals use to explain things. Psychological distance (PD) in turn affects individuals’ perception of CL, and then affects individuals’ perception and behavior. This provides an important theoretical basis for studying the choices that affect people’s behavior. Therefore, this study introduces CL theory into the field of sustainable development from the PD perspective, selects university students related to construction as the subject, and analyzes the underlying psychological mechanism that causes a separation between perception and behavior in sustainability among university students in AEC fields. This is of great significance for promoting the conversion of cognition to behavior, improving the level of sustainable development behavior of college students in the AEC field and promoting sustainable development in the field of architecture and the world.

## 2. Literature Review

### 2.1. Sustainable Development Related Research

Sustainable development has been an extremely important topic to the world [14,28,29]. The WCED defined sustainable development as “development which meets the needs of current generations without compromising the ability of future generations to meet their own needs” [28]. Because the concept of sustainable development involves a wide variety of fields—including resources, environment, ecology, and society—a variety of scholars have also defined sustainable development from different perspectives. Some scholars emphasize that development must be within the carrying capacity of resources and the environment. By maintaining the quality of natural resources and the services they provide, the benefits of economic development are maximized [9]. Human beings improve quality of life without exceeding the carrying capacity of the ecosystem that supports the earth [10]. Although the WCED definition has been criticized and questioned [29], it currently remains the most widely accepted. Therefore, we use this definition as the cornerstone of this study. The core of sustainable development is to handle the relationship between people and nature and to solve environmental problems in the development process.

Some scholars have engaged in research on AEC students’ sustainability from the perspective of education to promote sustainable development in the engineering field. Bhandari, et al. [30] pointed out that existing multidisciplinary courses on sustainable engineering have been expanded to include new courses, such as Sustainability Concepts and Tools, Sustainable Water and Waste Systems, Sustainable Energy Systems, Sustainable Agriculture and Food Systems, and Independent Module of the Sustainable Building System. From the environmental, economic, and social perspectives, Carew and Mitchell [31] pointed out that there are potentially significant differences in the sustainable behaviors that professors can advocate to undergraduates. Some scholars have tried game software to promote sustainable education for undergraduates in the engineering field. For instance, Dib and Adamo-Villani [32] assessed students’ attitudes towards sustainability and found that, compared with environmental majors, civil engineering students’ attitudes towards sustainability were less positive, and pointed out that early emphasis on sustainability could affect students’ awareness of the importance of environmental engineering through games.

### 2.2. Perception and Behavior Related to Sustainability Research

The discussion of the relationship between perception and behavior is of great interest in social psychology, whose main purpose is to investigate the predictability of perception on behavior. Sustainability perception refers to individuals’ knowledge, awareness, and attention to sustainable issues [33,34]. Locke [35] believes that all human behaviors are related to factors such as perception and emotion. Courbalay, et al. [36] found that perception does not only affect behavioral decision-making at the conscious level but also plays a role at the unconscious level, affecting individual behavior through mechanisms such as habits. Wossink and Van [37] even claim that the improvement of perception inevitably leads to reasonably expected behavior.

The results of most research in sustainable development confirm that perception determines behavior. Echegaray and Hansstein [38] and Zhang, et al. [39] have shown that increasing ones’ knowledge regarding recycling improves individuals’ behaviors. Cooke and Vermaire [40] showed a positive correlation between environmental knowledge and behavior. None and Datta [41] found that environmental knowledge has a significant positive correlation with the green purchasing behavior of Indian residents. Lee, et al. [42] also discovered that environmental knowledge has a significant positive effect on individuals’ environmental purchasing behavior and citizenship behavior. Studies have also shown that individuals’ environmental knowledge can significantly impact their environmental awareness, sensitivity, and attitudes towards the environment [43,44]. Furthermore, Kelly, et al. [45] pointed out that environmental knowledge affects one’s attitudes towards the environment and further alters environmental behaviors. Tadesse [46] showed through empirical research that awareness of the environment has a significant positive effect on household waste recycling.

Although plenty of studies have shown that perception has a positive effect on behavior, some scholars have also argued that there is a perception-behavior inconsistency. Maloney, et al. [47] studied the relationship between environmental knowledge and environmental emotions, verbal commitments, and actual commitments, and they found no correlation between environmental knowledge and the other three aspects. They ascribe that to broad environmental knowledge, difficulty to measure, difficulty to obtain environmental knowledge, and low environmental knowledge. Subsequent research also found that there is no relationship between environmental knowledge and environmental behavior [48,49], a weaker relationship between the two [50,51], or at most, a moderate degree of correlation [52]. Some scholars pointed out in their ABC model that environmental protection behavior is the result of a combined effect of individual psychological attitudes and external factors such as economic conditions, social structure, and systems, under which it is difficult to align environmental behavior with environmental awareness [53]. Wang [54] used annual cross-sectional data to conduct a statistical analysis of the inconsistency of public environmental awareness and behavior and found that public behavior is characterized by “merely talking about but not practicing” environmental protection. Lu [55] discovered that most people are aware of the harm of water pollution, but only a small number of people act when encountering environmental problems.

Wang, et al. [56] discussed five factors: policy, consumer efficiency, information factor, interpersonal communication factor, and marketing factor, and pointed out that the cultural cultivation of sustainable consumption should be strengthened. Wei, et al. [57] conducted a survey on risk perceptions, attitudes, and behaviors regarding climate change, and they found that risk perceptions and behaviors are inconsistent. Individuals are aware of the adverse effects of climate change on human health, but only a few are directly affected. Chen, et al. [58] noted that there are attitude-behavior differences in environmental sustainability behaviors in China and defined potential gaps from the perspective of attitude formation and transformation. Rausch and Kopplin [59] studied the purchase behavior of sustainable clothing, and consumers’ perceived aesthetic risk was found to have a negative impact on the intention-behavior relationship. Mousavi, et al. [60] found that there is no significant correlation between recycling knowledge and waste management behavior. Kang, et al. [19] used an econometric approach to empirically test the reason for differences in farmers’ willingness and behavior in recycling. Lin, et al. [61] introduced the governance context as a moderating variable to analyze the relationship between farmers’ psychological perceptions and their behaviors. Therefore, the relationship between sustainable development perception and behavior needs further research, particularly with regard to university students in the AEC fields.

Linden, et al. [62] put forward five insights from psychological science through practical examples, which can help the government improve public policy formulation related to climate change. They argue that policymakers should (a) emphasize climate change as a present, local, and personal risk; (b) facilitate more affective and experiential engagement; (c) leverage relevant social group norms; (d) frame policy solutions in terms of what can be gained from immediate action; and (e) appeal to intrinsically valued long-term environmental goals and outcomes. However, their study on psychological distance is limited to qualitative analysis, and there is no large sample verification, which makes the research results less credible. At the same time, they fail to provide deep insights on the various dimensions of psychological distance.

Similarly, in order to increase public participation in climate change, Jones, et al. [63] surveyed Australian residents. They employed Principal Component Analysis to quantitatively research the four psychological distance dimensions: geographic, temporal, social, and uncertainty. Research results suggest that climate communications framed to reduce psychological distance represent a promising strategy for increasing public engagement with climate change, in which Social Distance and Uncertainty play a leading role. However, this study only discussed the influence of psychological distance in the exact four dimensions and missed out research on the high and low interpretation level and the interaction between psychological distance and interpretation level.

Subsequently, Schuldt, et al. [64] attempt to offset the shortcomings associated with previous research by using concrete (or abstract) language to describe climate influences, aiming to study the role of spatial distance and the level of interpretation. However, the results showed that this reduced psychological distance did not translate into increased policy support. It is shown that the influence of psychological distance on public engagement in green practices has not been unanimously agreed. Nevertheless, the research was only carried out from a single spatial dimension, and whether the influence of other dimensions of psychological distances are also consistent with the conclusions of the research remains to be explored.

### 2.3. Research Gaps

The literature review has shown several gaps: First, there are still debates concerning the influence of psychological distance on public engagement in sustainable practices. Second, in terms of research content, these studies are all studying the public’s participation in sustainable development from the perspective of climate change. However, climate change is relatively far away from people, and the garbage problem is closely related to daily life. The research results between them are quite different. Therefore, the influence of psychological distance on the recycling behavior of university students is still an uninvestigated territory. Third, the other dimensions of psychological distance, such as information distance and experience distance, and the influence of each psychological distance dimension and their interactions with high and low construal levels need to be studied. Fourth, the subjects of these studies are the general public. As a special group of society and elites of the country, university students are in a critical stage of establishing correct value system and forming good behavior habits. Their words and deeds can greatly affect the development of the industry, but they have not received enough attention among scholars. Therefore, this research bridges existing research gaps by probing the six dimensions of psychological distance: time, space, social, hypothetical, information, and experience, as well as the interactive influence of different construal levels on the sustainable behavior of university students, and it identifies the key psychological distance dimensions. The research findings can provide a reference for the university programs in modifying their curriculum and pedagogical practices in order to transform student’s high perception to behavior and subsequently promote sustainable development.

**Figure 1:**
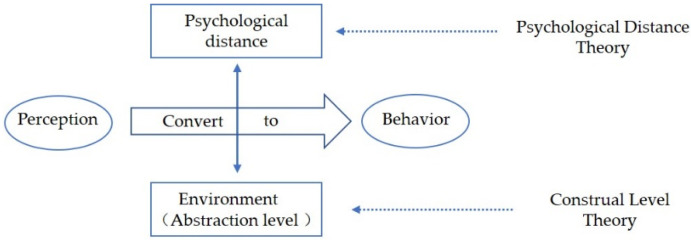
Research Conceptual Framework.

In AEC fields, most studies on the sustainability practices of university students begin with sustainability courses and educational settings. Although existing literature has greatly contributed to the body of knowledge regarding perception and behavior in sustainable development from a variety of aspects, little research has been conducted on the differences between sustainable development perceptions and behaviors on the part of university students in the AEC fields. Moreover, most existing research focuses on the external manifestations and influencing factors of the inconsistency between perceptions of sustainability and behaviors associated with it [47,48,49,50,51,52,53,54,55,56,57,58,59,60,61,62,63,64]. Many studies used correlation analyses, and situational simulations are rarely used to study the phenomenon [47,48,49,50,51,52,53,54,55,56,57,58,59,60,61,62,63,64]. Furthermore, there is a lack of in-depth analysis of the internal psychological causes of this phenomenon through empirical study. Additionally, the subjects of existing research into environmental perception and behavior mainly include the general public, with little attention paid to university students, especially those in AEC fields. Therefore, this study attempts to integrate CL theory and PD theory in an experiment designed to gain deeper insights into the perceptions and behaviors of university students in the AEC fields, probing the internal mechanisms of perception and sustainable behavioral decision-making of individual university students through empirical study based on situational settings. Hence, the research framework is shown as Figure 1.

## 3. Theoretical Bases

### 3.1. Construal Level Theory

CL Theory is a purely cognitive-oriented theory in social psychology [65]. Its core content is that people’s perception of events or objects depends on human mental representation of events. Human mental representations of cognitive objects have different levels of abstraction, that is, CL [66], which can be divided into two levels. High CL refers to a relatively abstract representation that is goal-oriented, independent of background information, relatively coherent, consistent, and contains the primary and decisive characteristics of the objects. Low CL refers to relatively specific representations, which are not goal-oriented, rely on background information, and include secondary and unique characteristics of the objects [67,68]. The CL depends on the PD that people perceive along with the cognitive object, which in turn affects people’s judgment and decision-making. When the PD of the cognitive event or object is far, one tends to use high CL to abstract the overall characteristics of objects. When the PD is close, one is inclined to use low CL with specific and local characteristics [69]. From the CL perspective, university students tend to adopt different representations in various situations, and then form different judgments and decisions relating to sustainability.

### 3.2. Psychological Distance

The concept of PD was first proposed by British esthetician Edward Bullough [70]. In 1988, Liberman, Nira and Yaacov [69] introduced PD to the field of social psychology for the first time and connected it with CL theory. From the CL theory perspective, PD refers to the individual’s direct experience with reference to the point of origin, the moment, the present, and the perception of when and where an event or object happened, who it happened to, and whether it occurs [69,70]. PD is comprised of four dimensions, namely temporal distance, spatial distance, social distance, and hypotheticality. Temporal distance refers to the time people perceive an event based on the present, such as the near future (one week later) or the long-term future (one year later). Social distance refers to the individual’s perception of the distance between the subject of the event and him or herself, much like the self and others. Spatial distance refers to the individual’s perception of the distance between events or objects, such as hometown and foreign countries. Hypotheticality refers to the likelihood of an event occurring. People make evaluations and decisions based on the time distance, spatial distance, social distance, and hypothetically different subjective experiences of objects or things relative to themselves, that is, the PD effect.

Liberman, et al. [71] pointed out that PD includes other dimensions in addition to the abovementioned four. Fiedler, et al. [72] believes that informational distance and experiential distance should also be used as dimensions of PD as they are also critical to perception and behavior. Informational distance refers to the amount of knowledge or related data an individual has regarding decision-making, that is, the number of facts, experiences, details, and knowledge units. The greater the information density, the shorter the distance. Experiential distance judges the effectiveness and amount of first-hand information (such as an individual’s own experience) or second- and third-hand information (communication with others or through other media). Therefore, to be reliable, this study selects six dimensions (see Table 1): temporal distance, spatial distance, social distance, hypotheticality, informational distance, and experiential distance, to study the behavioral decision-making of university students in sustainability.

## 4. Methodology

### 4.1. Research Design

This study mainly adopts an experimental research method to study the perception and behavior of university students in the AEC fields with regard to sustainability using situational simulation. Experimental study refers to the research method that is grounded in the principles and methods of scientific experiments, guided by theories and hypotheses. It purposefully manipulates certain factors or conditions to explore the causes and patterns of behavior and phenomenon changes.

**Figure 2:**
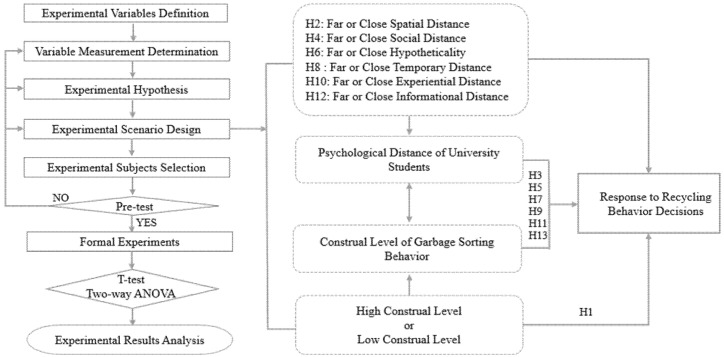
Research design.

This study employs the factorial experimental design method to collect empirical data, test the experimental hypotheses, and thus ensure the internal and external validity of the experimental research. To calibrate the experiment settings, this research first conducts a pretest using a series of specific experimental steps and content. First, it establishes the experiment’s research hypothesis and then, based on the research purpose, designs the experimental scenarios and tasks, which include designing a situational questionnaire to construct a real sustainable behavioral decision-making scenario context. By manipulating the six PD dimensions and CL, the descriptions of specific situational contexts of PD under different CLs were used to assess their impact on the sustainable behavior of university students in the AEC fields (see Figure 2).

For a rigorous and scientific analysis of the data obtained in the experiments, this study employs two-way ANOVA and *t*-test for data analysis. Once obtained, the experimental data are processed and analyzed to evaluate the hypotheses formulated in this study (see Equation (1)).

y_i_ = β_0_ + β_1_Z_1i_ + β_2_Z_2i_ + β_3_Z_1i_Z_2i_ + e_i_, (1)

where:

- y_i_ is the response to recycling of the ith subject;
- β_0_ is the coefficient for the intercept;
- β_1_ is the mean difference on CL;
- β_2_ is the mean difference on PD;
- β_3_ is the interaction of CL and PD;
- Z_1i_ is the dummy variable for CL (θ = high, 1 = ow);
- Z_2i_ is the dummy variable for PD (θ = close, 1 = far);
- e_i_ the residual for the ith subject.

### 4.2. Experimental Variables

Previous research has pointed out that peoples’ recycling behaviors are closely related to sustainability, which is regarded as key for sustainable development [73]. Therefore, this study uses the responses of college students to recycling to study the behavior of sustainable development. The experiment consists of CL, spatial distance, social distance, hypotheticality, temporal distance, experiential distance, informational distance, etc., as independent variables, and college students’ decision-making in recycling behavior as a response variable.

As for the manipulation of variable measurement of high and low CL, drawing on the definition of CL in previous studies [74], this experiment defines the abstract, general, and main trash environment scene description as the high CL. The specific, detailed, and marginal trash environment scene description is defined as the low CL. According to the characteristics of the university students and the environment in which the subject (experiment participants) of this study is located, the PDs of different dimensions are defined respectively, through repeated discussions with several professors, graduates, and undergraduates in the AEC fields. The behavioral decision-making of students’ recycling behavior is used as the experimental observation variable, and a five-point Likert scale is used to measure the willingness of behavioral decision-making in recycling (1—Very Unwilling, 2—Unwilling, 3—Moderate, 4—Willing, 5—Very Willing). The specific settings are shown in Table 2.

### 4.3. Experimental Hypothesis

Based on theoretical analysis of the CL and PD, as well as the experience and knowledge of experts, combined with the research objectives, this research proposes the following 13 hypotheses for the experiments:

**Hypothesis** **1** **(H1)**. *The main effect of CL on the recycling behavior of university students in AEC is significant*.

- Experiment (a) hypothesis:

**Hypothesis** **2** **(H2)**. *The main effect of the spatial distance of the waste crisis on the recycling behavior of university students is significant*.

**Hypothesis** **3** **(H3)**. *The spatial distance and CL of the waste crisis have a significant interaction effect on the recycling behavior of university students*.

- Experiment (b) hypothesis:

**Hypothesis** **4** **(H4)**. *The main effect of the social distance of the waste crisis on the recycling behavior of university students is significant*.

**Hypothesis** **5** **(H5)**. *The social distance and CL of the waste crisis have a significant interaction effect on the recycling behavior of university students*.

- Experiment (c) hypothesis:

**Hypothesis** **6** **(H6)**. *The main effect of the hypotheticality of the waste crisis on the recycling behavior of university students is significant*.

**Hypothesis** **7** **(H7)**. *The hypotheticality of the waste crisis and CL have a significant interaction effect on the recycling behavior of university students*.

- Experiment (d) hypothesis:

**Hypothesis** **8** **(H8)**. *The main effect of the temporal distance of the waste crisis on the recycling behavior of university students is significant*.

**Hypothesis** **9** **(H9)**. *The temporal distance of the waste crisis and CL has a significant interaction effect on the recycling behavior of university students*.

- Experiment (e) hypothesis:

**Hypothesis** **10** **(H10)**. *The main effect of the experiential distance of the waste crisis on the recycling behavior of university students is significant*.

**Hypothesis** **11** **(H11)**. *The experiential distance of the waste crisis and CL have a significant interaction effect on the recycling behavior of university students*.

- Experiment (f) hypothesis:

**Hypothesis** **12** **(H12)**. *The main effect of the informational distance of the waste crisis on the recycling behavior of university students is significant*.

**Hypothesis** **13** **(H13)**. *The informational distance of the waste crisis and CL have a significant interaction effect on the recycling behavior of university students*.

### 4.4. Pretest

In order to examine whether the experimental design is rational and whether the experiment’s manipulation is effective, this study first performed a pre-experiment to avoid potential waste and re-work due to improper experimental design and blindly carrying out formal experiments. In order to ensure the reliability and validity of the experiment, the research was carried out through two rounds of pre-experiments after multiple rounds of discussion and modification by the research team.

The pre-experiment was conducted in a quiet environment where there was little distraction, with suitable lighting and room temperature. Members of the research team gave subjects a brief introduction to the purpose of the experiment and obtained their informed consent. After the subjects agreed and were ready, the research team administered the experimental materials to the subjects. In the first round, four graduate students with research experience successively participated in the experiment under high and low CLs and different PD manipulations to avoid interference. The research team then recorded and analyzed their responses, interviewed the subjects regarding their feelings, asked them to share their thoughts regarding the situational settings, and requested feedback on the questions and modifications to the questionnaire. Due to minor flaws and defects found in the first round of pre-experiment, the design of the experimental situations was modified and improved accordingly.

In the second round of the pre-experiment, a total of eight undergraduates of different grade levels were divided into two groups of four subjects with a low and a high CL, and they were subjected to different PD manipulations. The subjects read the material regarding the situational settings, answered the questions, and were interviewed after completing the experiment questions. The following aspect were examined: whether each situation and the corresponding question were clearly expressed, whether there was any ambiguity, and whether the material was in line with the actual situation of university students in AEC. Combining preliminary test results, after discussing the controversial situational settings and topics, the research team modified or deleted problematic questions until a consensus was reached. The experimental design was then finalized.

### 4.5. Experiment Manipulation

**Figure 3:**
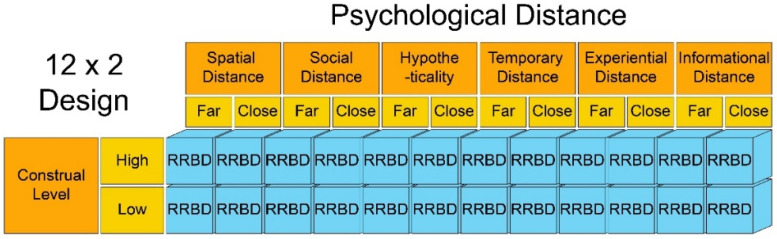
Illustration of the factorial experiment design.

The study first tested the degree of discrimination of the experiment’s situational settings and then conducted the interaction test on the CL and PD as two major factors. Finally, in order to test the impact of PD and CL on the individual recycling behavior of university students in the AEC fields, this study adopts 12 (PD: far and close) by two (CL: high and low) inter-group experiments, forming a total of 24 experimental scenarios (see Figure 3), measuring the cross combination of different factor levels to assess the impact of CL and PD on the recycling behavior of AEC university students.

## 5. Data

In order to ensure reliability of the experimental data and sample representativeness, this study adopted a snowball method by contacting close connections (faculty members in the field of AEC) at different universities and requesting they recruit their students to take part in the experiments. We then conducted simple training on the experiment and described the relevant situational settings to the data collectors. The research team made itself available throughout the entire experiment, ensuring someone was always on hand to answer any questions posed by subjects any time during the process. The subject matter of the experiment comprised civil engineering, real estate, construction management, cost engineering and other engineering-related fields, and architecture. Sample data involved 415 online participants and 141 offline participants (see Table 3), including undergraduates from all levels and graduate students pursuing master and doctoral degrees. Participants came from 20 different universities in 10 regions, including Beijing, Guizhou, Hebei, Guangdong, Guizhou, Shaanxi, Jiangsu, Shanxi, Shandong, Shanghai, and Tianjin, with different economic strata and distinct cultures. The universities and programs are quite different, including 1st-tier, 2nd-tier, and 3rd-tier schools. The main reason for gathering data from different universities and programs is to ensure sample representativeness so as to obtain reliable conclusions and inferences. The ratio of male to female, particular majors, and range of academic qualifications are shown in Table 3. A total of 556 samples were gathered, 514 of which were valid.

## 6. Results and Discussion

### 6.1. Analysis of the Perception and Behavior of University Students in AEC Fields

In order to corroborate initial observations and hypotheses, this research analyzes the relationship between sustainability perceptions and behaviors by collecting data on how students from different universities in the AEC fields across China understand the importance of sustainability and corresponding behaviors. Based on the collected experimental data and statistical analysis (see Table 4), the research shows that the average score of perception regarding the importance of sustainability is 4.53, the average score of willingness to recycle is 3.12, and the *p*-value of the *t*-test is 1.52 × 10^−141^ < 0.05, indicating that the research hypothesis is supported and that there is significant inconsistency between university students’ perceptions and behaviors in terms of sustainability. It shows that there indeed exists a “high perception, low behavior” phenomenon among university students in the AEC fields in China. Based on this finding, the study then focused on gaining deeper insights into the psychological influence mechanism behind the cognitive and behavioral differences through psychological experiments, exploring the root causes of their formation.

### 6.2. The Influence of Multi-Dimensional Psychological Distance on Behavior

Before analyzing the influence of multi-dimensional PD on behavior and in order to ensure the reliability and accuracy of the experimental scenario settings, this study collected data on the opinions of participants in the experiment regarding the effectiveness of PD in the scenario design. The descriptive statistics of the results in Table 5 suggest that the average scores of the discrimination evaluation on the PD situational setting were mostly greater than 2.5 (based on a 0–4 Likert scale), and the median and mode were both greater than or equal to 3, indicating that all participants thought there was a significant difference between far and close scenario settings of the PD. The definition and manipulation of PD in these experiments was therefore deemed effective and valid.

In this study, the two factors of CL and PD are analyzed together to gain insights into their individual influence on the subjects’ recycling behavior and the interaction between the two factors. The six dimensions of PD on participants’ sustainable behavior were then analyzed individually. Two-Way ANOVA was performed to achieve the objectives based on the empirical data. Table 6 shows that the influence of PD on the decision-making involved in the recycling behavior of university students was significant, that is, there was a statistically significant difference (F = 11.21, *p*-value = 9.05 × 10^−11^ < 0.001), which means the six dimensions of PD had a significantly different impact on participants’ recycling behavior (F = 39.65, *p*-value = 2.00 × 10^−16^ < 0.05), but CL had an insignificant impact on university students’ recycling behavior (F = 0.93, *p*-value = 0.35 > 0.05). The interaction between CL and PD (F = 0.14, *p*-value = 0.98 > 0.05) was also not significant. Therefore, hypothesis H1 has not been supported by the empirical data gathered in this study.

This result indicates that, regardless of whether the situations’ wording is concrete or abstract, it has no significant impact on the subjects’ recycling behavior. This is probably due to the inadequacy and ineffectiveness of sustainability education in Chinese universities because many schools offer no courses in sustainability, and a few universities offer only one or two introductory courses, so that most students have only a vague awareness of the importance of sustainability and have not yet transferred such awareness to their daily behaviors. On the other hand, the phenomenon of divergence of knowledge and behavior is also closely related to the inadequacy of recycling hardware, such as too few recycling bins or inconvenient deployment of recycling bins at many Chinese universities, and the rigidity and discontinuity of software, such as managerial methods. For example, many university campuses lack recycling facilities, and recycling bins are often placed at locations that deviate greatly from students’ routes between classrooms and dormitories or gathering places. In many cases, students need to take trash from the seventh floor to the first floor where the recycling bins are placed. Sometimes, recycling facilities are lacking or are too small for sites that have the largest flows of people. These are likely the causes inhibiting students’ recycling, which collectively contribute to the research findings.

### 6.3. Influence of Single-Dimensional Psychological Distance on Behavior

The influence of each of the six dimensions of PD on subjects’ recycling decision and its interaction with CL was further analyzed through two-Way ANOVA, and was shown to be not significant according to the test statistic (F = 0.07, *p*-value = 0.79 > 0.05). This means the wording of the experiment’s situational settings and distance between the subject and recycling facilities did not impact each other’s influence on the decision of the subjects. The results of Experiment (a) (see Table 7) confirm that CL has no significant main effect on participants’ recycling decision because the test statistic is F = 0.76 (*p*-value = 0.39 > 0.05), that is, the description of the experiment settings does not affect participants’ recycling actions. However, spatial distance has a significant main effect on participants’ recycling decision as the test statistic F = 17.82 and *p*-value = 2.68 × 10^−5^ < 0.001, that is, the distance of the recycling facilities from the participants affects their recycling decision to a great extent. Therefore, H1 and H3 are not supported, and H2 is verified by the research data.

The results of Experiment (b) (see Table 8) confirm that the main effect of CL on the experiment object’s waste recycling decision is not significant based on the test statistic (F = 0.02, *p*-value = 0.88 > 0.05). The interaction between CL and social distance on participants’ waste recycling decision is not significant according to the test statistics (F = 0.15, *p*-value = 0.70 > 0.05). The main effect of social distance on participants’ recycling decision is significant in terms of the test statistic (F = 72.65, *p*-value = 2 × 10^−16^ < 0.001), that is, the relationship between the subjects and the event or object affects their recycling decision to a great extent. Therefore, H1 and H5 are not substantiated, and H4 is supported by the empirical data.

The results of Experiment (c) (see Table 9) suggest that the main effect of CL on participants’ recycling decision is not significant according to the test statistic (F = 0.25, *p*-value = 0.62 > 0.05). The interaction between CL and the experiential distance on participants’ waste recycling decision is not significant according to the test statistic (F = 0.14, *p*-value = 0.71 > 0.05). Hypotheticality has a significant main effect on participants’ recycling decision according to the test statistic (F = 17.82, *p*-value = 7.40 × 10^−9^ < 0.001), that is, the likelihood that participants believe a serious incident would occur significantly affects their recycling decision. Therefore, H1 and H7 are not supported, and H6 is supported by the research data.

The results of Experiment (d) (see Table 10) reveal that the main effect of CL on participants’ recycling behavior is not significant due to the test statistic (F = 0.108, *p*-value = 0.74 > 0.05). The interaction between CL and temporal distance on participants’ recycling decision is not significant based on the test statistic (F = 0.21, *p*-value = 0.65 > 0.05). The main effect of temporal distance on participants’ recycling decision is significant (F = 23.99, *p*-value = 1.15 × 10^−6^ < 0.001), suggesting that the time an event happens to participants significantly affects the participant’s recycling decision. Therefore, H1 and H9 are not supported, but H8 is substantiated by the research data.

From the results of Experiment (e) (see Table 11), the test statistics confirm that the main effect of CL on participants’ recycling decision is not significant (F = 0.48, *p*-value = 0.49 > 0.05) and the interaction between CL and experiential distance on participants’ recycling decision is not significant (F = 0.01, *p*-value = 0.93 > 0.05). The experiential distance has a significant main effect on participants’ recycling decision according to the test statistic (F = 34.71, *p*-value = 25.47 × 10^−9^ < 0.001), that is, whether the participant witnesses the event (first-hand information) greatly affects his/her recycling decision. Therefore, H10 is supported by the research data but H1 and H11 are not.

The results of Experiment (f) (see Table 12) verify that the main effect of the CL on participants’ waste recycling decision is not significant according to the test statistic (F = 0.02, *p*-value = 0.90 > 0.05). The interaction between CL and information distance on participants’ decision-making regarding recycling is not significant in terms of the test statistic (F = 0.03, *p*-value = 0.86 > 0.05). The main effect of information distance on participants’ recycling decision is significant according to the test statistic (F = 51.96, *p*-value = 1.23 × 10^−12^ < 0.001), meaning the frequency of information reception affects participants’ decision regarding recycling to some extent. Therefore, H12 is supported by the research data but H1 and H13 are not.

The experiment results confirm that the language of the situational settings and PD do not interfere with each other. Additionally, all individual PD dimensions have a significant effect on AEC students’ recycling decision. The results imply that students’ recycling behavior is affected by the spatial distance between the students and recycling facilities, the relationship between students and places facing the waste crisis, students’ belief in the likelihood that a waste crisis will emerge, time when the waste crisis will emerge, and the source and frequency of the information. The findings make great sense because university students represent a highly educated group, meaning they generally have suitable knowledge and social responsibility and tend to recycle trash if it does not take too much effort or time. People intuitively prioritize situations according to their relationship to the situation and the urgency felt. Most reasonable people are greatly influenced by information they think is reliable, and first-hand information is generally viewed as more reliable than second- or third-hand information. The frequency of one’s exposure to information also deeply impacts behavior and decision-making.

### 6.4. Comparison of the Influence of Single-Dimensional Psychological Distance on Behavior

The experiments above show that PD affects the recycling decision of university students in the AEC fields. Although the six dimensions of PD are related to each other, there are differences in their links, and some dimensions are more critical than others [74]. In order to obtain deeper insights into the level of influence of different dimensions of PD, this study further conducts a set of pairwise *t*-tests on the recycling decision of university students under different dimensions of PD. Table 13 shows that spatial distance is significantly different from all other PD variables. Informational distance is significantly different from social distance and spatial distance, and temporal distance is significantly different from social distance and spatial distance, but experiential distance is not significantly different from the other four variables. The influence of each dimension of PD on the recycling decision of university students is ranked in terms of mean response scores: spatial distance (Ms = 4.07), informational distance (Mi = 3.91), temporal distance (Mt = 3.89), experiential distance (Me = 3.86), hypothetical distance (Mh = 3.84), and social distance (Mso = 3.76). It is shown that spatial distance is the most influential dimension of PD, followed by others, to affect university students’ decision to recycle trash.

## 7. Conclusions

University students in AEC fields are the main force and future leaders of the construction industry, and their values deeply shape the model and direction of the industry’s future development. In addition, university students’ perceptions and behaviors profoundly affect the words and deeds of their subordinates and colleagues. Therefore, grounded by SCT, PD, and CL theory, this study attempts to gain insight into university students’ perception of sustainability and their recycling behavior. Experiments with the designed settings that incorporated PD dimensions were used to analyze the impact of PD and CL on recycling behavior decisions. The results of this study can improve perceptions of sustainability on the part of university students in the AEC fields, promote university students’ recycling behavior, and provide theoretical support for formulating sustainable development policies.

This study shows that there is an inconsistency between perceptions and behaviors among university students. The impact of PD as a whole on recycling behavior is significant. Moreover, the six dimensions of PD taken individually also have a significant impact on participants’ recycling behavior. However, CL has no significant impact on recycling behavior nor is its interaction with PD significant. Further comparisons among influence levels of university students under different dimensions of PD show that the six PD dimensions exhibit varying impact on students’ behavior (in descending order): spatial distance, informational distance, temporal distance, experiential distance, hypothetical distance, and social distance.

This study contributes to the body of knowledge by introducing CL and PD into research regarding sustainability perceptions and behaviors in the construction industry because an understanding of them can help identify root causes for the inconsistency found between AEC students’ perceptions and behaviors concerning sustainability. Specifically, the major contributions include: 

(1) The introduction of psychological distance and construal level theories into sustainable development cognition and behavior is an important contribution to enriching the application of construal level theory and psychological distance theory. The current academic research on sustainability cognition and behavior consistency is mainly from ABC Theory, Theory of Planned Behavior (TPB), Theory of Reasoned Action (TRA) [8], and other studies on sustainable development cognition and behavior, but they mostly focus on external factors, such as policies and environmental protection knowledge. However, for any event or object, as long as it is not self, immediate, and local experience, it inevitably undergoes a change in psychological distance. Even if the gap between cognition and behavior of sustainable development is affected by psychological distance, the inner psychological mechanism of the difference between cognition and behavior of sustainable development is still unclear. The construal level theory abstracts people’s mental representations of cognitive objects or events into different levels of interpretation, using underlying logic to explain things in the world, and few scholars have used them to study differences in cognition and behavior. Therefore, this study uses psychological distance and construal level theories to explore the reasons for the differences in cognition and behavior of sustainable development. The root causes for the difference in behavior provide a theoretical basis for improving the level of sustainable development, as well as a new perspective for related issues and future research.

(2) The current psychological distance theory is not perfect. Whether there are other critical dimensions is still controversial [9]. In addition to the four dimensions (i.e., time distance, social distance, spatial distance, and hypothesis) that are generally recognized and applied, there are other distance dimensions (i.e., information distance and experience distance) that will also affect people’s thinking and behavior [9]. However, the existing consensus of these psychological distance dimensions has not been supported by extensive experimental research, and it needs to be further explored [9,10]. At the same time, each psychological distance dimension affects people’s behavioral decision-making to different degrees. However, the current research on psychological distance mostly conducts parallel studies on each dimension without comparing the degree of importance, which is not conducive to solving problems for the key influencing dimensions. Therefore, in this study, experience distance and information distance are included in the psychological distance dimension to conduct experiments and to compare the importance of the psychological distance of each dimension, and to deepen and improve the research on psychological distance to a certain extent, and it is also one of the theoretical contributions of this research.

(3) Applying the experimental method to sustainable development cognition and behavior by incorporating psychological theories in the design is another contribution of the research method. Most of the related research is to construct models and conduct research through interviews, questionnaires, and other methods. However, these methods have certain limitations: the respondents are easily affected by the surrounding environment, which leads to results quite different from reality. As an important method in the field of cognitive psychology, experimental method can simulate the actual situations through experimental environment design, concept analysis, and operation guidance, so that the subjects can be immersed in the scene, which can stimulate the subject’s true feelings, leading to more realistic and reliable decisions.

(4) In terms of research content, on one hand, currently scholars mostly focus on sustainable materials and new buildings in AEC industries. Few scholars conduct research from cognition and behavior. On the other hand, some research on cognition and behavior has mainly focused on the general public. University students’ values can shape the model and direction of the industry development because they are the backbone and future leaders. Studying university students in a certain sense is more meaningful than studying the general public because university students’ perception and behavior change can not only affect their own behavior but also their colleagues and subordinates. However, little attention is paid to this special group. Therefore, this study explores the sustainable development cognition and behavior of AEC college students, which is of great significance to promote the transformation of college students’ cognition of sustainable development to behavior and improve the level of sustainable development.

## Figures and Tables

**Table 1:** Experimental variable definitions.

PD	Definition
Temporal Distance	Refers to how far or near the time people perceive the occurrence of an event or object.
Spatial Distance	Refers to the individual’s perception of the geographical distance between events or objects and oneself in space.
Social Distance	Refers to the perception of the individual’s self-based, event subject or object’s distance from space to space, such as self and others.
Hypothetical Distance	Refers to the probability of the occurrence of the subject or the distance from reality.
Informational Distance	Refers to the amount of knowledge or related data related to decision-making.
Experiential Distance	Refers to the effectiveness of first-hand information or second- and third-hand information.

**Table 2:** Situational settings of experimental variables.

Variable		Situational Settings
CL	CL-Low	Take-out boxes, plastic bags, wastepaper, and leftovers. Other waste is piled up; fruit, bread, and other foods rot and mold; there is a rat infestation; and mosquitoes and flies’ breed. All dormitory doors and windows must be closed tightly, and students must wear masks when passing by/in close proximity to the setting.
	CL-High	The environment is messy, smelly, and unsightly.
PD	a. Spatial Distance	The location of the waste crisis: “room” vs. “downstairs”.
	b. Social Distance	The relationship between the entity of the waste crisis and oneself: “self” vs. “others”.
	c. Hypotheticality	The probability of the occurrence of a waste crisis: “90% believe” vs. “10% believe”.
	d. Temporal Distance	The time when the waste crisis occurred: “now” vs. “one year later”
	e. Experiential Distance	Effectiveness of obtained information on the occurrence of waste crisis: “witness it with my own eyes” vs. “news report”.
	f. Informational Distance	How often is the perceived waste crisis: “daily” vs. “occasionally”.
Behavioral Decision		Are you willing to recycle trash based on the situational settings?

**Table 3:** Summary of demographic data.

Item	Category	Count	Percentage
Sex	Male	337	65.6%
	Female	177	34.4%
Major	Civil Engineering	40	7.8%
	Real Estate	3	0.6%
	Construction Management	343	66.9%
	Cost Engineering	106	20.7%
	Architecture	3	0.6%
	Other	18	3.5%
Undergraduate	Freshman	177	43.5%
	Sophomore	87	21.4%
	Junior	101	24.8%
	Senior	42	10.3%
Graduate	Master	95	89.6%
	Doctoral	11	10.4%

**Table 4:** *t*-test of students’ perceptions and behaviors.

	Perceptions	Behaviors
Mean	4.53	3.12
Variance	0.36	0.86
Pooled Variance	0.61	
df	1070.00	
*t* Stat	29.65	
*p* (T <= *t*) two-tail	1.52 × 10^−141^	
*t* Critical two-tail	1.96	

**Table 5:** Descriptive statistics of responses to the hypothesized situations.

	Spatial	Social	Hypothetical	Temporal	Informational	Experiential
Mean	2.32	2.51	2.52	2.39	2.55	2.63
Median	3.00	3.00	3.00	3.00	3.00	3.00
Mode	3.00	3.00	3.00	3.00	3.00	4.00
SD	1.34	1.29	1.26	1.36	1.28	1.25
Variance	1.78	1.68	1.59	1.84	1.63	1.57

**Table 6:** The results of two-way ANOVA for CL and PD Effect on recycling behavior.

	Df	Sum Sq	Mean Sq	F Value	Pr (>F)	
CL	1	1	0.78	0.93	0.35	
PD	5	47	9.43	11.21	9.05 × 10^−11^	***
Individual PD	6	200	33.36	39.65	<2.00 × 10^−16^	***
CL: PD	5	1	0.12	0.14	0.98	
Residuals	5238	4408	0.84			

Signif. codes: 0 ‘***’.

**Table 7:** Experiment (a) spatial distance vs. CL.

	Df	Sum Sq	Mean Sq	F Value	Pr (>F)	
CL	1	0.60	0.64	0.76	0.39	
Spatial Distance	1	15.10	15.10	17.82	2.68 × 10^−5^	***
CL: Spatial Distance	1	0.10	0.06	0.07	0.79 *	
Residuals	872	738.70	0.85			

Signif. Codes: 0 ‘***’ 0.01 ‘*’.

**Table 8:** Experiment (b): social distance vs. CL.

	Df	Sum Sq	Mean Sq	F Value	Pr (>F)	
CL	1	0	0.02	0.02	0.882	
Social Distance	1	68.50	68.52	72.65	<2 × 10^−16^	***
CL: Social Distance	1	0.10	0.14	0.15	0.70	
Residuals	872	822.50	0.94			

Signif. Codes: 0 ‘***.

**Table 9:** Experiment (c): hypotheticality vs. CL.

	Df	Sum Sq	Mean Sq	F Value	Pr (>F)	
CL	1	0.20	0.21	0.25	0.62	
Hypotheticality	1	29.20	29.22	34.10	7.40 × 10^−9^	***
CL: Hypotheticality	1	0.10	0.12	0.142	0.706	
Residuals	872	747.40	0.86			

Signif. codes: 0 ‘***’.

**Table 10:** Experiment (d): temporal distance vs. CL.

	Df	Sum Sq	Mean Sq	F Value	Pr (>F)	
CL	1	0.10	0.09	0.11	0.74	
Temp. Distance	1	19.90	19.89	23.99	1.15 × 10^−6^	***
CL: Temp. Distance	1	0.20	0.18	0.21	0.65	
Residuals	872	722.90	0.83			

Signif. codes: 0 ‘***’.

**Table 11:** Experiment (e): experience distance vs. CL.

	Df	Sum Sq	Mean Sq	F Value	Pr (>F)	
CL	1	0.40	0.41	0.48	0.49	
Experience Distance	1	29.20	29.22	34.71	25.47 × 10^−9^	***
CL: Experience Distance	1	0.00	0.01	0.01	0.93	
Residuals	872	734.20	0.84			

Signif. codes: 0 ‘***’.

**Table 12:** Experiment (f): informational distance vs. CL.

	Df	Sum Sq	Mean Sq	F Value	Pr (>F)	
CL	1	0.00	0.01	0.02	0.90	
Informational Distance	1	38.20	38.23	51.96	1.23 × 10^−12^	***
CL: Informational Distance	1	0.00	0.02	0.03	0.86	
Residuals	872	641.60	0.74			

Signif. codes: 0 ‘***’.

**Table 13:** Descriptive statistics and *p*-values of the pairwise *t*-test of PD variables.

	Mean	Sta. Error	Median	Spatial	Experiential	Informational	Hypothetical	Social	Temporal
Spatial	4.07	0.03	4.00	-	-	-	-	-	-
Experiential	3.86	0.03	4.00	7.40 × 10^−6^	-	-	-	-	-
Informational	3.91	0.03	4.00	0.0009	0.3148	-	-	-	-
Hypothetical	3.84	0.03	4.00	1.30 × 10^−6^	0.6833	0.1624	-	-	-
Social	3.76	0.03	4.00	6.80 × 10^−11^	0.0644	0.0023	0.1461	-	-
Temporal	3.89	0.03	4.00	0.0002	0.5484	0.6730	0.3275	0.0100	-
